# Foreign Medical and Physical Science and Literature

**Published:** 1818-10

**Authors:** 


### FOREIGN MEDICAL AND PHYSICAL SCIENCE AND LITERATURE.

TO exalt, in an unmerited degree, the importance of the discoveries and improvements in science, of the existing period, and to lament the ignorance and prejudice of former times, have ever been trite and fertile subjects, for observers of the character and manners of the age. But, in the greater part of those effusions of exultation, an ebullition of vanity, and a want of proper reflection on the powers of the human intellect,—joined, perhaps, with a degree of laudable enthusiasm,—are more evident than cool and rational observation. It would appear, in many instances, that those observers have been so dazzled with the surrounding light, that the shades behind them appeared utter darkness; and that the horizon they beheld, was considered the boundary of human knowledge.

To extol the acquisitions which have of late been made in medical science, may, perhaps, be falling into the error we have been deprecating; but may not the philosophical mode of induction with which the study of it is now pursued, lead us to hope that such an imputation is not to be feared? Many circumstances have concurred to give a new character to the medical knowledge of the present era.

In the dark ages of modern times, and even until a very late period, the little literary intercourse that existed between the different civilized nations of Europe was not sufficient to excite national emulation: indeed, prejudice tended rather to repress than cherish so fertile a source of improvement. Thus, we find, the discoveries and opinions of HUNTER were for many years unknown beyond the coasts of our island; and, when they were noticed in other countries, it was with a degree of apathy that appears incompatible with reason, much less with that ardent love of knowledge and truth which should characterize philosophers. The sparing manner in which genius is distributed would not permit any nation, alone, to make great advances. Men of talents might observe and store up important facts; but they were added to the collected mass of error; their characteristics were obscured, and their application perverted. The few original geniuses that appeared, disdaining to follow guides who were constantly leading them astray, took new courses, and obtained followers; but encountered no rivals able to execute the difficult task of pointing out their aberrations from reason, or of shewing the mazes in which they had trod, from being guided by a vivid imagination, not directed by the hand of judgment: none who could determine whether truth or error lay hid under the splendid veil which Genius throws around his offspring. In proof of this, we have only to consider, how many systems have been raised, and obtained whole nations as votaries, which would not have existed for a day, had they been investigated by persons unbiassed by national affection. Indolence, also, tended much to favor this submission to the authority of others: it was indulged by the refuge it could take in a sanctuary already erected, instead of performing the laborious task of collecting what appeared to be mere atoms; and which were continually escaping, for want of knowledge to adapt them to their proper use. But a new era in medical philosophy has arrived, and now

> "The glow-worm 'gins to pale his ineffectual fire,
> And we scent the morning air."

It is to the free intercourse existing between philosophers of all nations, the universal emulation thence excited, and the cautious manner in which new opinions are examined, that the character of philosophical induction which distinguishes medical literature of the present day may doubtless be attributed.

These reflections have made us determine to devote to foreign medical science a greater share of our attention than we have hitherto done; it being evident that we shall by that means more perfectly fulfil the professed object of our work,—that of conveying to our readers information of whatever may tend to contribute to our stock of useful knowledge.

We shall commence this department with the consideration of a highly interesting work,—the first volume of *the Transactions of the Physico-Medical Society of New York.* This is a society lately instituted, with the same object that has given origin to numerous others, from which the practice of medicine has derived so many advantages. Societies of this kind must be particularly useful in the United States: where, as in all nations rapidly advancing in civilization, and enjoying a great degree of political liberty, so many bold and original geniuses appear. The truth of this observation will be demonstrated by a consideration of the papers contained in the volume before us. It commences with—

*An Introductory Discourse on the Sophistication of Medical Theory; by* Elias Marks, M.D.

To give an analysis of a production, of which every sentence expresses ideas so intimately connected with the whole, that, to isolate any one, would be to break that connected chain of reasoning on which the communication of knowledge so intimately depends,—must be universally allowed to be impossible; our readers will therefore excuse our passing over this paper, to those of more practical importance.

*Observations on certain Causes which Influence the Decarbonising Function of the Lungs; by* Charles E. Pierson, M.D.

There are, we believe, few physicians, accustomed to reflection and actual observation of disease, who do not think that the generality of pathologists of the present day have fallen into error, by almost entirely neglecting the doctrines of the humoral pathology. Considering disease to consist in deranged action alone, the state of the fluids has been too much neglected. The secretions in disease, it must be acknowledged, have been examined with the most scrupulous attention; but it has been merely in reference to the morbid action supposed to be productive of the change.

The essay of Dr. Pierson we consider valuable, from its directing our attention to variation from the natural state, in the qualities of the blood, as the cause of disease; and containing many original remarks, which will probably lead to the knowledge of some important facts, both in physiology and pathology. Although we consider there is some truth in the opinions of the author, we are not disposed to concur with him in their full extent, but think that many of the facts he has adduced to illustrate them, may be referred to other causes.

Dr. Pierson commences with stating the conclusions drawn by Messrs. Allen and Pepys from their experiments on respiration; which we transcribe for the convenience of our readers, as they are the ground-work on which the hypothesis of the author is raised.

"1st. The inspired air imparts none of its oxygen or nitrogen to the blood.

"2dly. The blood loses a principle, viz. carbon; which, by its union with the oxygen of the inhaled air, forms carbonic acid gas.

"3dly. The watery vapour found in expired air is the serous discharge from the surface of the bronchial tubes.

"4thly. The blood derives heat from the decomposition of the inspired air; all the latent heat of the oxygen gas not being necessary to the formation of carbonic acid gas.

"5thly. The dark colour of the venous blood is owing to its being surcharged with carbon; and the bright scarlet colour of the arterial blood to its parting with carbon in the process of breathing."

Dr. Pierson observes, that it would appear from these facts that the principal design of respiration is to separate from the blood a matter, which, if retained in any considerable quantity, is extremely deleterious to life. This leads to the statement of the principle of his hypothesis—"That there are certain circumstances affecting respiration, which subject the human system to a morbid retention of the carbon of the blood, and thereby produce derangement and disease." The perfect or imperfect decarbonization of the blood obviously depends upon one of the three following circumstances: viz. the velocity of its circulation, the state of the atmosphere inhaled, and the freedom with which the air is admitted to the extreme pulmonary vessels. He first considers, "*The effects of neglect of exercise on the excretion of carbon from the lungs.*" It appears, from the experiments of Lavoisier, Sequin, and others, that, during exercise, there is a great augmentation in the discharge of carbon from the lungs in respiration. Sequin examined his own respiration in a state of rest, and found that 1344 cubic inches of oxygen gas were consumed per hour. After brisk exercise for a quarter of an hour, it was found that he consumed oxygen at the rate of 3200 cubic inches per hour, which is 1856 cubic inches more than in a state of rest. Now a volume of carbonic acid gas is precisely equal in bulk to that of the oxygen gas which is required for its formation; consequently, in this experiment, there was separated from the blood, in exercise, as much more carbon per hour than is evolved during rest, as enters into the composition of 1856 cubic inches of carbonic gas. Dr. Pierson observes that, taking these facts for granted, it cannot be considered altogether fanciful to suppose, that protracted inactivity of body should be productive of some inconvenience to the system, solely from an undue accumulation of carbon. If this accumulation of carbon be denied, it must be on the supposition that its admission into the circulation by the lacteals is diminished, exactly in proportion to its excretion from the lungs. This precise balance, the author thinks, cannot exist. If then, he observes, a surcharge of carbon exist, may we not infer, from our knowledge of the consequences of the more obvious retentions of this principle, that a morbid action of the system must result; and that the long train of affections that follow a sedentary life may be ascribed to this cause? The experiments of Bichât seem to shew, that a similar condition of body is induced by a redundancy of carbon in the circulation. Many facts, Dr. P. thinks, tend to prove, that the effects of carbon, in unusual quantities, are deadly sedative. He notices the healthy and vigorous state of body of those persons who have a capacious thorax; and, in opposition to this, the meagre and half-animated frame of the unfortunate *puer cœrulus*. Among the disorders arising from neglect of exercise, may be mentioned *obesity*. Fat is known, by chemical analysis, to be composed of four-fifths of carbon, and the remaining part of oxygen and hydrogen. This fact, the author considers, tends much to favor his opinions.

*The effects of the rarefied state of the air on the quantity of circulating carbon* are next considered. The rarefied state of the air we breathe may depend either on its high temperature or on a diminution of weight in the atmospheric column. A correspondent division, in treating of its consequences, must necessarily be followed.—The *effects of a heated atmosphere* are first noticed. Experiments have shewn, that the quantity of oxygen gas consumed by a man in an hour, when breathing air of the temperature of fifty-four degrees, was found to be 1344 cubic inches: when the air was raised to seventy-nine degrees, only 1210 cubic inches were consumed in the same length of time; consequently, a high temperature of the atmosphere must be productive of a retention of the carbon; and to this cause, Dr. Pierson is disposed to attribute many of the diseases, experienced on residing in a warmer climate than that to which a person has been accustomed. He however considers that there are other emunctories, by which part of the carbon is conveyed out of the system, when there is so great a failure in the functions of the lungs: those are the skin and liver. There are many facts, he observes, which lead us to believe, that the liver is concerned in this office. Heat greatly increases the secretion of the bile; bilious complaints are endemic in all hot countries. In the production of the mortal epidemic and endemic bilious fevers of hot countries, miasmata may be chiefly concerned; but the heat may be the sole agent in giving the disease its bilious character. Dr. P. adduces the following facts, as tending to favour the opinion, that the liver co-operates with the lungs in decarbonizing the blood.

1st. *The bile abounds in carbon.*

2d. *The bile is secreted from the venous blood.* All the secretions, except the bile, are formed from arterial blood. The intention of this has been a source of much speculation. Chemists have discovered, that the blood in the vena porta contains a greater proportion of carbon than that of any other part. The reason assigned for this is, that, the blood in the porta coming from the viscera of the abdomen, no change has taken place at the extreme vessels, like that which takes place on the skin ; which he thinks, as we have before stated, assists in decarbonizing the blood. In the fœtal state, it is probable the liver entirely supplies the place of the lungs. The meconium is, beyond doubt, produced by the liver. By chemical analysis it is found closely to resemble the adult bile, and to consist chiefly of carbonaceous matter. It is said, that the blood returned from the placenta by the umbilical vein is more florid than that in the umbilical arteries. This may be the case, and yet the liver perform the office of lungs; for the decarbonization may be but in part executed in the lungs, while the liver does the remainder, and perhaps the chief of it. Dr. Pierson proposes the following questions respecting the fœtal liver:—

Why does the liver secrete a bilious matter in the fœtus, when it can answer no purpose in the intestines, for they are not brought into action until after birth? Why is the liver of the fœtus so over proportioned in size to the rest of the body? Why is the blood of the umbilical vein made to circulate through the substance of the liver, before it reaches the heart, and is distributed over the whole system?

The author then treats of *the effects of rarified air from diminished weight of the atmospheric column.* This affects the decarbonizing function, from the rarified air not supplying so great a portion of oxygen to the lungs, in a given time, as it does when of greater density and pressure. The debility, languor, and headache, experienced by many persons in a light air, is attributed to the above cause. When the blood is not sufficiently decarbonated in the lungs, it is retarded in the extreme pulmonic vessels; and its flow back to the heart is slow and languid. The uneasiness thus excited is productive of the asthmatic paroxysm.

*The imperfect decarbonization of the blood from mucous obstruction in the lungs* is lastly treated of. In pituitous asthma, the blood of the patient is usually dark; his circulation slow and feeble; there is a great deficiency of bodily strength; the heat is much below the common temperature; and the patient experiences, in a degree, all that variety of distress and infirmity, to which the unfortunate blue boy is a victim. These are considered as the consequences of a want of proper decarbonization of the blood; but what is more in point, the author observes, is the colour of the matter expectorated, which is frequently of a black colour. Peripneumonia is attended with more debility, and is more apt to sink the system into a low debilitated state, than pleuritis, or other local inflammations. May not this arise from the obstruction which the mucus, lining the bronchial surface, affords to the necessary decarbonization of the blood? The author concludes with observing, that this subject was primarily contemplated with a belief that there were truths lurking in it, which had not been investigated; and, if they are not rendered conspicuous in this essay, the opinion of the writer has not been weakened by the inquiry.

*Observations on Cynanche Laryngea, with Cases;* by James C. Bliss, M.D.

We shall briefly notice this essay, notwithstanding the accurate description and useful practical remarks it contains. But the able manner in which the subject was treated by Dr. Farre, in a paper published in the third volume of the Transactions of the Medico-chirurgical Society, will prevent the necessity of our considering it in detail, on the present occasion. The disease is an highly important one, and, previously to the treatise which we have mentioned, it had not been accurately described by any writer; its peculiar symptoms may therefore be unknown to some of our readers, for the benefit of whom, we shall give a general account of them, as described by Dr. Bliss.

The disease commences with uneasiness in the larynx, hoarseness, and febrile symptoms: at the accession, a slight difficulty is also experienced in swallowing; and, if the fauces be examined, there will be a slight appearance of inflammation. As the symptoms progress, acute pain is felt posterior to the thyroid cartilage; the respiration becomes difficult and wheezing; the articulation indistinct and hoarse; deglutition becomes at length extremely difficult, or altogether interrupted,—the patient being in danger of suffocation whenever it is attempted: on examination at this time, the epiglottis will appear to stand erect, and not to close the rima glottidis, which explains this difficulty. The febrile symptoms are now much increased; the face becomes turgid; the pulse is greatly increased, both in force and frequency; and the heat of the body is greater than natural. This stage of the disease is not of very long continuance: the respiration becomes still more difficult and distressing; the powers of life begin to decline; the pulse diminishes in strength; the heat becomes less; the features assume a ghastly appearance, and the patient dies of suffocation.

From this very brief description of symptoms, it will appear evident, that this disease is not referable to any species of Cullen's genus, cynanche; and a more manifest difference is exhibited, in the dissection of those who have fallen victims to it. The epiglottis is found much enlarged, from the previous inflammation; there is sometimes effusion of serum beneath the membrane covering it, sometimes so as to prevent its closing the glottis; a thickened state of the mucous membrane covering the larynx may also be observed, with considerable effusion of serum and coagulable lymph beneath it; this sometimes to such an extent, as to close the rima glottidis. The upper part of the pharynx and fauces, and the muscles of the larynx also, exhibit signs of previous inflammation. General and local blood-letting, emetics, blisters, and the warm bath, should be fully employed in the treatment of this disease. In cases in which suffocation is to be feared, the operation of tracheotomy may be resorted to with more probability of success than when it is performed in croup, in which the inflammation generally extends into the minute ramifications of the bronchiæ.

*Observations on the Efficiency of Emetics in Spasmodic Diseases; with an Enquiry into the final Cause of Sympathetic Vomiting;* by Joseph M. Smith, M.D.

Every practitioner must have suffered the painful task of being obliged to set by the bed-side of a patient during the paroxysms of spasmodic diseases, to witness the inefficacy of all the remedies usually recommended for their relief. Indeed, in the greater number of instances, with the exception of opium and venesection, they are worse than useless; and the latter remedy is too seldom and sparingly employed. Although the use of emetics in those diseases is by no means new, yet much praise is due to Dr. Smith for his attempt to make it more general, and for his judicious remarks on the administration of them, and their mode of action. The superiority of emetics, as remedies in the treatment of convulsions, consists, 1st, in the facility with which they may be administered; 2dly, in the certainty of their operation; 3dly, in the speedy manner in which they check the fits; and, 4thly, in their producing, ultimately, no disagreeable effects. The tartarized antimony is the medicine which should be used. If the paroxysms quickly succeed each other, it should be given in divided doses during the intervals. As soon as nausea takes place, the spasms will be found less violent, and the patient will complain of languor; as it increases, the spasms become still weaker, and the intermissions longer; and, when full vomiting occurs, the paroxysms cease to return. Should the patient be unusually plethoric, and especially if there be great arterial excitement and a determination to the head, it will be proper to take some blood from the arm, previously to giving the emetic. When the paroxysms manifest a disposition to return, a dose of laudanum will, in general, prevent them.

The exciting causes, the author observes, of hysteria and epilepsy are perhaps as multifarious as those of any other disease; but, however various they may be, no discrimination is necessary in determining on the employment of emetics, provided there is energy enough in the system to withstand their debilitating operation, and there is no organic affection.

The mode of action of emetics may be referred to the *sympathy*

existing between the stomach and the rest of the body; from which, during the state of nausea, a general relaxation of the system, and more particularly of the muscular fibres, takes place; a state opposite to the violent action which accompanies convulsion. This is exemplified, by the facility with which a dislocated limb may be reduced, if nausea approaching to vomiting, be previously produced by tartarized antimony.

Those observations led Dr. Smith to enquire, whether there is any foundation in nature for the use of emetics as antispasmodics; and that there is, he thinks, may be established by the following facts. The passage of a calculous through the biliary ducts is accompanied with vomiting: this is considered as the effect of the *vis medicatrix naturæ*; the object of which is to relax the duct, that it may more easily pass. The same circumstance attends the passing of a calculus through the ureter. The colic is also usually accompanied with vomiting. The nausea attending the first stage of a paroxysm of fever is also adduced to illustrate this opinion; and the doctrine of Cullen, of spasm on the extreme vessels, which it is intended to relieve, is considered powerfully to support it. Why vomiting should generally occur, when certain parts of the abdominal viscera require relaxation, or are affected with spasm, and only occasionally, when the muscles of voluntary motion are convulsed, may be explained, by supposing that, as an effort of the vis medicatrix naturæ, it is more readily and actively excited by morbid action in organs essential to life, than by disease in parts comparatively unimportant.

The vomiting with which women are affected, during the first months of utero-gestation, is referred to the same object. To suppose that the embryo is alone a sufficiently distending power, would be fanciful and nugatory; inasmuch as it is evident, that if the womb had not a disposition or aptitude to enlarge, it would expel the ovum as certainly as it does coagula of blood; and how could it be otherwise, since it is a law of muscular parts, to contract whenever a distending force is applied to them. Dr. Denman expressed an opinion, "that this distension is evidently not mechanical from the enlargement of the ovum, but from the accession of a new principle; for the uterus is never fully on the stretch, like a bladder inflated with air, but relaxed in such a manner as to be apparently capable of bearing the increase of the ovum without inconvenience." That the dilatation of the uterus is not entirely passive seems to be proved by the fact, that, in all the instances of extra-uterine gestation which have been examined, dilatation of the uterus to a considerable extent had taken place. The vomiting, during the early stages of parturition, and the ceasing of it, when the os uteri has become fully dilated, is a fact which tends much to support the opinion of Dr. Smith, respecting the influence of vomiting on this organ.

We shall conclude this article in our next Number.

### *Physiological and Medical Researches on the Causes, Symptoms, and Treatment, of Gravel;* by F. Majendie, M.D. &c. &c.

There is no disease, for obtaining an accurate knowledge of which, the abilities of the chemist, the physiologist, and the practical physician, are more requisite, than that of calculous concretions in the kidnies and bladder: a work, therefore, on this subject, by M. Majendie, must be considered with no small degree of attention by the medical profession.

"Notwithstanding its frequency, (says the author,) the distressing symptoms with which it is accompanied, and its serious consequences, gravel has been but slightly noticed in general works on medicine and surgery: in particular treatises on the diseases of the urinary organs, it is considered in a very imperfect manner, and, to my knowledge, it has not hitherto been the professed subject of a work."*

M. Majendie first treats on the nature of the sand and stones voided by persons affected with this complaint. The knowledge of the composition of those substances is entirely due to the researches of modern physiologists. Van Helmont, notwithstanding his penetrative imagination, could only compare their formation to the deposition of tartar from wine. Paracelsus believed them to be formed of *animal resin*, hardened by what he termed the *spirit of urine.* It is to Scheele that we are indebted for the origin of our knowledge on this subject: he shewed that the concretions voided with the urine were principally formed by a particular acid, to which he gave the term *lithic*. The researches of Wollaston, Fourcroy, Vauquelin, Brande, and Marcet, have fully confirmed the discoveries of that illustrious chemist, and have furnished us with a knowledge of many other important facts.

Some species of calculi contain a small proportion of oxalate of lime; and, in a few instances, they are almost entirely formed of oxalate or phosphate of lime. When they remain long in the kidnies, they become covered with a thin layer of phosphate of lime and magnesia. M. Claubry has lately found in the kidney of a man four calculi, each formed of a nucleus of oxalate of lime, and an exterior layer of uric acid. Upwards of thirty specimens of gravel were analysed by M. Majendie, and he found them all composed of uric acid united with a small proportion of animal matter.

The urine of man, he observes, and of all animals who use a diet of aliments containing a large proportion of azote; as flesh, fish, eggs, &c. contains uric acid; and it is very abundant in that of those who live exclusively on animal food. On the contrary, in animals who are fed with vegetables, the urine does not contain the smallest quantity of uric acid.

* Dr. Marcet, in his treatise on Calculous Disorders, has not thought it necessary to separate the history of gravel from that of other calculous formations in the urinary passages.

Water, at the temperature of from sixty-five to seventy degrees of Fahrenheit, dissolves only the $\frac{1}{1720}$ part of its weight of uric acid; boiling water dissolves it in the proportion of $\frac{1}{1150}$, and the solution, on cooling, deposits a portion of the acid in thin laminæ. The solubility of the salts formed by uric acid, is relative to that of their bases. Almost all the acids are capable of decomposing them.

The cystic oxide, the author states, very rarely enters into the formation of gravel and urinary calculi; but, as the nature of this animal matter is not generally known, it may be proper to mention its general properties. Calculi formed of cystic oxide, are semi-transparent, of a yellowish colour, and have a lustre similar to that of bodies of a density powerfully refractive. Exposed to heat, in a retort, they furnish carbonate of ammonia of a fetid odour; there passes also an heavy fetid oil, such as is obtained from animal matter, but in a much less proportion than that which results from a distillation of uric acid. These properties shew, that, like uric acid, it is principally composed of azote; it is therefore probable, that it is produced by the same causes which determine the formation of uric acid. This substaucc is but very slightly soluble in water, not at all in alcohol, or the acetic, tartaric, and citric acids; it is, on the contrary, soluble in the muriatic, nitric, sulphuric, phosphoric, and oxalic acids, as well as in potash, soda, lime-water, and the carbonates of potash and soda. The greater number of those properties approach to those of uric acid.

When treating on the causes of gravel, M. Majendie observes, it is evident the formation of uric acid is not accidental, or owing to diseased action, but that it is one of the essential principles of the urine of a person in a state of health: it is then, however, held in solution by the urine; but, in this disease, it is deposited in the passages intended for the collection and evacuation of that fluid. As water, at the temperature of 80°, dissolves the $\frac{1}{1800}$ part of its weight of uric acid; the urine of a person in a state of health, being about 30°, may be supposed capable of retaining in solution about the $\frac{1}{1500}$ part of its weight, supposing, what nothing yet known tends to refute, that the other principles of the urine do not favour its solution. This being granted, it appears that three evident causes may diminish, in a manner absolute or relative, the dissolving property of the urine in respect to the uric acid.

1. An increase in the quantity of the uric acid, the quantity of urine remaining the same, or not increased in proportion to the acid.

2. A diminution in the quantity of the urine, that of the uric acid continuing the same, or not diminishing in proportion to the urine.

3. A diminution of the temperature of the urine, whether its quantity or nature continue the same or undergo the changes above mentioned.

Among the causes which augment the proportion of uric acid, a too nourishing diet, and particularly of animal substances, may be considered the most efficient. When, with this, persons lead a sedentary life, or use exercise which requires but very little exertion of the muscular system, as is the case with literary men, the rich, and old people in general, the disposition to its formation is much increased. As long as the urine is sufficient to hold in solution the uric acid formed, any increase in its quantity is productive of no inconvenience; and this is doubtless what happens to many persons who use the diet just described, and who are not affected with gravel; but, as soon as the increase of the quantity of urine does not proportionally follow that of the acid, gravel is immediately formed.

In general, the proportion of urine is relative to that of the liquids drank; and, therefore, a person who uses a full diet of animal food, will be more likely to escape gravel, if he drink a large, than if he drink a small, quantity. This must be understood, if the fluids he takes be not charged with alcohol.

Animals which live on vegetable food, void a much larger proportion of urine than carnivorous animals, notwithstanding they drink much less. All causes which are known to diminish the quantity of urine will, therefore, favour the formation of gravel; as an abundant cutaneous transpiration, sweats, and lying much in bed, will consequently tend to produce it. A diminution of animal heat, and consequently of the temperature of the urine, will have the same effect, and this is one reason why old people are so particularly subject to it. Various other causes will also favour its production; it appears from the observations of Dr. Scudamore,* that the poor people of a district between Tunbridge Wells and Lewes, who drink much of "hard beer," are particularly afflicted with it. There are many persons, who, after violent exercise, to which they have not been accustomed, pass large quantities of gravel. It frequently follows indigestion. M. Majendie does not consider the formation of gravel, in these cases, the consequence of indigestion, but that they both arise from the same cause. The author is inclined to attribute the freedom the Indians enjoy from gravel, not to the climate, as some have done, but to their living on vegetable food. The results of the enquiries of Dr. Marcet respecting the relative proportion of patients with stone, in the different hospitals of Europe, favours this opinion. At Guy's Hospital, the proportion is one in three hundred; at St. Bartholomew's, one in three hundred and forty; in the Royal Infirmary at Edinburgh, only one in a thousand. This difference in the proportion between the Scotch and the English may be explained by the diet of the poor of the former country being almost entirely of vegetables, while the latter take a large quantity of animal food. The notion that calcareous waters tend to produce gravel is erroneous; they, on the contrary, may be advantageously employed for the relief of this complaint. M. Majendie considers

---

* Related in his Treatise on Gout.

that the causes which tend directly or indirectly to produce the formation of gravel may be reduced to the following:—

1. Mature and old age.

2. A too nutritious diet, principally composed of aliments containing a large proportion of azote.

3. Want of bodily exercise, lying too long in bed, &c.

4. The habit of drinking but little.

5. The use of generous wines and spirituous liquors.

6. Abundant cutaneous transpiration, copious sweats, and serous evacuations, in persons previously disposed to gravel.

7. The bad habit of retaining the urine long in the bladder.

8. Particular causes, of which it is impossible to mistake the effects; although their mode of action may not be understood.

M. Majendie thinks that, under certain circumstances, a large quantity of gravel may be formed in a few hours, the solidification of the uric acid taking place as soon as the urine is secreted. He is not decided where this precipitation of the uric acid principally takes place, but supposes it to be in the pelvises of the kidnies.

Sand, in general, passes without causing much uneasiness, when the secretion of urine is copious; but, when otherwise, particles becoming joined together, form small calculi, which occasion great pain in their passage through the ureters. If any of those become arrested in their progress in the urinary passages, they form a nucleus for the formation of larger stones.

The following are the curative indications in this disease:—

To diminish the quantity of uric acid formed in the kidnies.

To augment the secretion of urine.

To prevent the solidification of the uric acid, by saturating it with a more soluble basis.

Gravel and calculi being formed, to favour their evacuation, and attempt their solution.

The formation of uric acid depending on the use of aliments containing azote, the mode of fulfilling the first indication is obvious; the quantity of animal food, eggs, &c. should be diminished, and, in some instances, entirely changed for a vegetable diet. This will generally correct the disposition to the formation of gravel. M. Majendie has seen cases, in which the patients had been for many years tormented by gravel and small calculi, entirely relieved in a few weeks by the use of a diet of sugar alone: this, however, cannot long be persisted in, as it disagrees with the stomach. A diet composed entirely of pastry, the farinaceous legumens, rice, potatoes, rye-bread, with a moderate proportion of sugar, has, in general, been found equally efficacious.

The most simple mode of increasing the quantity of urine is to drink plentifully; and this is the usual custom of persons afflicted with gravel. Many, by this means, without change of regimen, have been entirely relieved from the disease. The drink may be either simple water or infusion of some vegetable; mineral waters, &c.; sometimes nitre may be added with advantage. Five or six pints of drink daily will not be too large a quantity, particularly if the gravel be abundant. But little wine or spirituous liquors should be taken; and, when the gravel is considerable in quantity, a diet principally of vegetables (which favours a copious secretion of urine) should be employed.

The third indication, *to saturate the uric acid*, is to be effected by the alkalies or their sub-carbonates. That many substances are carried from the stomach to the kidnies, without passing through the general circulation, M. Majendie thinks is sufficiently proved: and of those, some of the saline preparations are most quickly transmitted, and in the most unaltered state. The alkaline sub-carbonates are decomposed in the urine, the uric acid unites with the base, and forms salts more soluble than the acid. This greater degree of solubility is in proportion to the excess of the base: it will, therefore, be necessary to preserve an excess of alkali in the urine, which may be known by the urine not presenting acid qualities.

The last indication,—*to favour the expulsion of sand and calculi, and attempt their solution*,—is not less, and is indeed frequently more, important, than either of the preceding. The expulsion of sand is usually not attended with much difficulty. The facility with which the particles are displaced, from their smallness, and their property of resting suspended in the urine, provided it contains a little mucus, concur to favour their evacuation: thus, in the greater number of cases, a certain quantity of aqueous drink will effect their evacuation with little difficulty. The expulsion of calculi will also be much favoured by drinking copiously of water. M. Majendie recommends emetics in these cases, considering that the pressure of the abdominal muscles during the action of vomiting will contribute much to hasten the passage of the calculi to the bladder and through the urethra. Emetics will also be beneficial by correcting the disordered state of the digestive organs which usually accompanies it. When the distress is very great, the most rigorous diet, general blood-letting, leeches, cupping, baths, and fomentations, are the principal measures to be adopted. If these are not effectual, exercise on foot and on horseback, if the patient can bear it, and the repeated use of emetics, should be resorted to. When it appears that the calculus has entered the bladder, but has not passed through the urethra, the measures before advised to prevent its increase should be employed. When the calculi are composed of uric acid, both chemical and physiological reasoning would lead us to suppose that those measures would be effectual. But this will not be the case when they are formed of the ammoniaco-magnesian phosphate, the oxalate of lime, &c. these, however, but rarely constitute the calculous concretions of patients afflicted with gravel.

Cystic oxide, as it contains a large proportion of azote, probably owes its formation to the causes which produce uric acid; the same remedies will therefore be advisable.

Acids have been proposed to be employed, in the treatment of gravel, consisting of phosphate of lime; the urine in cases of this kind contains an excess of ammonia, which is probably the principal cause of its production; and Mr. Brande has related a case in which carbonic acid was very beneficial. But M. Majendie could never effect the solution of depositions of this kind, when out of the body, by either the vegetable or mineral acids. He, therefore, thinks the best mode of treatment is to produce an abundant secretion of urine, and endeavour to relieve the distress and debility which usually accompany this disease.

---

*Pathological Observations illustrative of several Points in Physiology.* A Thesis presented to, and defended before, the Faculty of Medicine of Paris; by F. Lallemand, M.D. resident Surgeon at the Hôtel-Dieu, &c.—4to Paris, 1818.

Physiology, from its affording so wide a scope for contemplation and the exercise of a fertile imagination, has generally been a principal object in the studies of men of talents and original genius. But the investigation of it has been too much confined to the functions of animal bodies in a state of health; the axiom of Hippocrates "Νομιζω δὲ ὅτι περι φυσιος γνῶναι τι σαφὲς ὐδαμοθεν ἄλλοθεν ἔσται ἢ ἐξ ἰητρικης;"* has either not been sufficiently attended to, or the correctness of it not admitted. The advantages that physiology may receive from pathology are, perhaps, little less than those which medicine has derived from the cultivation of the former. Thinking thus, and considering the talents of M. Lallemand, and the opportunity afforded for the exertion of them, by his situation as resident surgeon at the Hotel-Dieu; we take up this work with no small degree of interest. It commences with some *observations relative to generation.* The subject which gave origin to them, was a case of extra-uterine gestation.

A woman, thirty-five years of age, of an irritable habit of body, and of the temperament M. Hallé has characterized by the term *uterine* "*lassata viris nedum satiata;*" about the beginning of October, was surprised *in coitu* with her husband, by a neighbour who suddenly entered the room. She was much agitated, and passed a very restless night after this occurrence; the following day she experienced a fixed pain in the left iliac region. She passed by the vagina a clot of blood, the evacuation of which was preceded and followed by a mucous discharge, apparently mixed with a little blood. About three months after this, the pain, which at the commencement had been confined to the left iliac region, now extended over the whole abdomen, which had increased in size: the evacuation of the fæces was attended with considerable pain. In this state she was admitted into La Pitié, on the 20th of January. M. Geoffrey, under whose care she was, considered her case as chronic peritoneal inflammation, with organic disease of some of the abdominal viscera, and treated it with aperi-

---

* De Veteri Medicina, c. 36.

ent medicines, the warm bath, &c. The pain continued to increase, she became much emaciated, and died on the 30th of March.

On opening the abdomen, the capillary vessels of the intestines, and the peritoneum covering the parts about the pelvis, were so distended with blood, as to lead to a supposition that violent inflammation had existed. The uterus had risen above the pelvis, and was about twice the usual size; on exposing its cavity, a soft, pulpy, retiform, reddish-coloured substance, of about a line in thickness, was found lining the whole of its inner surface, to which it was united by a tender flocky membrane. There was no opening into its cavity, either at the os uteri or the fallopian tubes. A spongy mass adhered to the left ovary, and to the broad ligament of the same side, and extended thence to the sigmoid flexure of the colon, and to the posterior surface of the uterus, being covered anteriorly by the inferior part of the great epiploon. This spongy mass resembled in some parts the membranes formed in inflammation; other portions were so similar in appearance and structure to a placenta, that it was immediately compared to it by all who were present. On turning aside the omentum and colon, a bag filled with water was discovered, in the midst of which floated the feet of a fœtus, the body and head being situated in the pelvis. It appeared to be of about the sixth month, was plump and well formed, except the head, which was flattened by the obstacles which had been opposed to its development; the umbilical cord, eleven inches in length, was inserted at the edge of the placenta by several large vessels; the chorion and amnios, perfectly distinct and easily separable from each other, lined almost the whole of the cavity of the pelvis. Opposite to the attachment of the placenta, the peritoneum was so injected with blood, that it appeared of a blackish colour, and some of its vessels were as large as a crow's quill.

M. Lallemande thinks, that conception had taken place previously to the period at which she received the shock to her feelings; and that the general relaxation and irregular action consequent on this state put a stop to the erethism of the fallopian tubes; and thus the ovum on its escaping from the ovary, not encountering the conductor which should transmit it to the uterus, had fallen into the cavity of the abdomen.

The substance found in the uterus was evidently the deciduary membrane. M. Chaussier has remarked, that he always found this membrane in the uterus, in extra-uterine gestation. Meckel and Weinknecht have made the same observation. Thus, it is proved, that the epichorion is not a membrane proper to the ovum, but formed in the uterus; the membrane which joined the chorion to the peritoneum was similar in appearance to the deciduary membrane. This membrane resembles those produced by adhesive inflammation, except that it is somewhat thicker and more vascular. The formation of the epichorion may, therefore, be considered as the consequence of the turgescent state of the uterus after conception which is very analogous to inflammation. The author then enters into the consideration of the mode in which the communication between the mother and the fœtus is established. The opinions that it is by a direct continuation of the vessels of the uterus with those of the placenta, and that which attributes it to a termination of the vessels in follicles between the uterus and placenta, are very satisfactorily controverted. M. Lallemande considers that this communication is precisely the same as that which takes place in adhesive inflammation; and thus it is as effectually established on any vascular surface to which the ovum may attach itself, as in the cavity of the uterus; the ovum irritates those parts, an effusion of lymph takes place, which becomes organised, and the circulation between them is effected in a few hours.

Some remarks ensue on *the Communication between two Placentas united into a Mass in some Cases of Twins.* M. Lallemand asks, Do the vessels of both the fœtuses communicate with each other? and, in this case, can we suppose that, during life, the blood of one may be mingled with that of the other? The greater number of authors on this subject, founding their opinions rather on reasoning than experience, deny the continuity of vessels. Smellie, however, injected the whole of a placenta common to twins through one umbilical cord. And, in later years, Chaussier, Breschet, Béclard, and Lebreton, have effected the same; but the passing of injections is no certain indication that a free communication existed during life. This fact has, however, been proved by some cases which occurred to the observation of the author.

In one instance, after cutting the umbilical cord on the birth of a child, the blood was found to spring from the placental end of the funis, just in the same manner as it does from a small artery after amputation. The cord was tied, and in a short time another fœtus was expelled. The two placentas were united into a common mass, although the membranes attached to them were merely contiguous: one of the cords was inserted in its centre, the other at its circumference. No attempt was made to inject it, because it was torn; but it appeared that there was not only a free communication between the two fœtuses during life, but also that this communication was by means of large vessels, since the blood issued in a stream from the end of the umbilical cord.

In another instance, which occurred to an eminent professor of midwifery, this fact was evinced in a manner which shews of how much importance it is that it should be generally known. This gentleman has made the case public with a degree of frankness that cannot be too much admired. Attending a woman in labour, he observed, after the birth of a living child, that another remained in the uterus; but, occupied with the infant, he did not examine the end of the funis which was attached to the placenta. His attention was soon directed to it by violent convulsive motions made by the fœtus remaining in the womb, which gave the mother considerable pain; they, however, ceased in a few instants. The head had descended into the lower part of the pelvis, and the use of the forceps appearing indicated, they were immediately applied without difficulty. This fœtus was as large and well formed as the former, but it was entirely without blood; attempts were made to restore it to life, but without success: it had lost all its blood from the end of the funis belonging to the other fœtus. The two placentas were united; one of the umbilical cords was inserted in its centre, the other at a little distance from the edge.

These cases, M. Lallemande observes, will show the importance of tying the maternal end of the navel string in cases of twins. This is a practice which, we believe, is universally adopted by the greater number of English practitioners of midwifery.

*Observations on the Functions of the different Parts of the Nervous System.*

This division of the work commences with some highly interesting and important observations on the functions of *the great sympathetic nerve.*

A woman, about forty years of age, in her sixth pregnancy, was admitted into the Hotel Dieu: she had passed through the five preceding with little inconvenience, and the children were all born living and healthy; but she had suffered much during the last: her constitution, which had been robust, was very seriously deteriorated; her skin had become transparent, and of a straw colour; serous effusion had taken place in the cellular membrane generally; the abdomen was enormously distended, which arose both from the extraordinary size of the uterus, and a dropsy of the abdomen. She could not lie horizontally without being threatened with suffocation. She calculated (judging from the time at which she had felt the motions of the fœtus) that she was in the eighth month of pregnancy. Two days before her confinement, she observed that she felt the motions of the fœtus distinctly, but that they were less powerful than usual.

When labour commenced, the pains succeeded each other rapidly, and the membranes soon became ruptured. To give an idea of the immense quantity of water they contained, it is observed, that after having passed through the bed and mattress, it flowed to a great distance about the room. The fœtus was soon after expelled suddenly, without having encountered hardly any resistance. From this occurring unexpectedly, M. Lallemande could not ascertain whether it had shown any signs of life immediately after its birth. It was a male child, without cerebrum, cerebellum, or spinal marrow; its flesh was firm, and the skin abundantly covered with down; the epidermis was no where detached; the external appearances of the body could not assist in determining its age, for a large quantity of firm red fat filling all the cellular tissue, gave all the parts a bulk disproportionate to their length. It is probable that the mother was right, in stating it to be eight months, for the testicles were found at the external part of the inguinal ring, ready to descend into the scrotum. All the parts of the face had acquired an extraordinary size, particularly the inferior jaw, which projected much beyond the upper; the eyes were large and projecting; the mouth open, and the tongue, very large, hung over the lower lip; the skin of the cranium, distinguishable by the long and thick hairs with which it was covered, terminated at the distance of an inch from the eye-brows; it afterwards diverged to each side towards the ears, diminishing in size, and ending in a point on a level with the last vertebræ of the back; the exterior edge of it was continuous with that of the forehead, neck, back, and loins; no mark of a cicatrix could be discovered; its interior edge circumscribed an irregular oval space, which extended from the middle of the base of the cranium to the sacrum, and from one scapula to the other. This want of skin was supplied at the upper parts, by the remains of the arachnoid membrane and pia mater, and, throughout the whole length of the vertebræ, by the dura mater, which, instead of forming a cylindrical cavity, was spread out as a flat surface, as were the spurious apophyses of the vertebræ; so that there was neither vertebral canal nor cranial cavity. These membranes had contracted adhesions with the skin by means of true cicatrices.

The transparency of the dura mater permitted the spinous apophyses to be distinguished through it, the separation of which formed, throughout the whole length of the back, a kind of groove, from seven to eight lines in length; on the surface of this membrane were two rows of whitish tubercles of the size of a pin's head, corresponding in situation with each intervertebral space: to these tubercles were joined the nerves of the neck, back, and loins, (the origin of those nerves had been destroyed with the spinal marrow.) On turning aside the dura mater, after having divided it, these nerves were seen leaving that membrane to pass out at the intervertebral spaces. The remains of the arachnoid membrane and the pia mater formed, behind the base of the cranium, a kind of cowl, which descended to the lower part of the back; below those membranes, the carotid and cerebral arteries, surrounded by veins, formed a net-work, in the midst of which the falx of the dura mater was discovered, and small isolated portions of brain, without any communication with nerves. All the nerves which arise from the brain were loose and floating at the base of the cranium.

The œsophagus and pharynx formed a kind of doubling, like a portion of intestine in an hernia; its cavity was considerably distended by a collection of matter, which on examination proved to be meconium. A little before its union with the stomach, the cavity of the œsophagus became very small in diameter, and indeed obliterated, so that the smallest stilette could not be passed on to the stomach. M. Lallemande observes, that in all the analogous cases he had read in authors, he regretted they had not examined the nerves with sufficient care; in this instance, he dissected with the greatest attention all the most important branches. Those of the face were in the natural state. The cervical nerves were very slender at their origin, but became of their natural size after having passed the intervertebral spaces a little distance; those of the superior extremities and thorax were of their usual size. The trunk of the great splanchnic nerve was as large as the median of the same fœtus; it arose from the largest ganglion, and terminated in the solar plexus, which gave to the hepatic, renal, pulmonary, coronary, and stomachic plexuses, filaments almost as distinct and large as those of an adult. On the right side, the branches furnished by the thoracic ganglions were, as well as those ganglions, less large and numerous. The nerves of the limbs offered nothing particular, neither did the bones or muscles of those parts. The stomach, drawn by the œsophagus, (the length of which was diminished two-thirds by the fold which it made at the upper part,) was pulled into the thorax; six or seven inches of the small intestines had also followed the stomach into that cavity; the mesentery and the rest of the small intestines were much lengthened; the cœcum, instead of occupying the right iliac cavity, had taken the place of the mesentery along the middle of the vertebral column; the right kidney had descended to the place of the cœcum; the spleen was hidden under the diaphragm, which was so forced upwards under the ribs as to lessen by one half the cavity of the chest. The cause of all these displacements, it is evident, was the diminution of the length of the œsophagus. The great intestines were filled with meconium, so similar to that found in the œsophagus that it was not possible to distinguish them.

With respect to the skeleton, the disorder the malady had produced in the ossification was confined to the cranium and spine. The bones of the cranium were separated aud turned over on the surrounding parts, in the same way as the spinous apophyses; those of the basis of the skull were not included in this derangement.

M. Lallemand makes some original and very judicious reflections on this interesting subject; but our limits oblige us to defer the consideration of them until the next number.

---

A deviation from the general distribution of the organs of the human body which is worthy of consideration, was lately observed in the Royal Hospital at Cadiz.

A man, twenty-four years of age, of an athletic form and tall stature, was received into the hospital for a wound between the seventh and eighth ribs, on the left side, of about half an inch in length. The instrument with which it was inflicted passed in a direction from below upwards, and a little from left to right. A thick fluid of a greyish colour escaped from the wound. The pulse became small, hard, and frequent; the respiration hurried; and there were great anxiety and vomiting. He was bled to a small amount, which was followed by an abatement of the symptoms; but they soon returned with their former serious character, and death shortly ensued.

The body was examined after death by Dr. Benjameda, the professor of Anatomy to the Royal College of Medicine and Surgery at Cadiz.

The left cavity of the thorax was much larger than the right,

The mediastinum was pressed towards the right side, from which, and the great bulk of the liver and the unusual projection upwards of its concave surface, the right cavity was rendered very small in its dimensions; which was filled with the right lung, much diminished in size. The left lung was so small, that it descended anteriorly, only to the lower edge of the third rib ; posteriorly, to the fourth. The concavity of the bases of both lungs was very considerable. The heart was situated perpendicularly behind the sternum; it did not rest on the diaphragm, being about an inch distant from it; so that it was suspended by the great blood-vessels. The stomach was divided into two portions: one, situated in the thorax, represented a membranous bag, larger than, but similar in figure to, the urinary bladder ; filling almost the whole of the left cavity of the chest. This membranous sac traversed this cavity in an oblique direction, and was divided inferiorly in two parts: one of which passed through a preternatural opening in the diaphragm, and was connected with the abdominal portion of the stomach; the other, forming a large *cul de sac*, rested on the diaphragm. The paries of this portion was very thick, and adherent to the pleura and surrounding organs. The abdominal portion of the stomach was small, and occupied the left part of the epigastrium ; it was of an irregular form, and appeared like a small entire stomach, placed perpendicularly: it formed two curvatures to right and left; the cardia was situated about an inch from the pylorus. The œsophagus, having passed the natural opening in the diaphragm in the usual manner, turned to the left to join the abdominal portion of the stomach. A kind of ring performed the office of a valve at the cardiac orifice, to prevent the return of food into the œsophagus. An oval opening in the diaphragm, half an inch in diameter, bordered by a tendinous circle, permitted the thoracic portion of the stomach to pass through that muscle.

No information could be obtained respecting the general state of the digestive and respiratory functions. The man appeared, however, to have had good health, and acted as pilot to a vessel.

---

An hospital has been established at Stockholm, under the direction of Mr. Osbeck, for the cure of the venereal disease. This gentleman, formerly surgeon of a small hospital at Wadstena, saw, during his residence at Copenhagen, Professor Winslow treat venereal disease by a *very rigorous diet*, which forms an indispensible part of the treatment, and by extract of hemlock. Convinced of the efficacy of this method, M. O. repeated the experiments in Denmark on some patients, and latterly in Sweden; but, instead of employing extract of hemlock, he made use of the extract of chærophylum silvestre. He was led to employ it, because he observed the apothecaries of Copenhagen commonly gathered this plant for hemlock ; he announced his discovery, demanded a commission for the purpose of proving the efficacy of his remedy, and undertook to reveal his secret for a stipulated reward. The request was acceded to, and Mr. Osbeck published a book in the Swedish language, entitled, An Exposition of the Method of curing inveterate Venereal Disease, in which his master, Winslow, is passed unnoticed. The most essential part of the treatment consists, according to Mr. Osbeck, in the use of the extractum chærophyli, and a low diet in an extreme degree.

All recent or inveterate venereal affections may be treated by these means; but it more particularly suits those cases where mercury has been employed without success, or where there exists a complication with scrofula or gout. The regimen of the patient, during the first six weeks, consists daily of five ounces of lean roast meat, and six ounces of bread. Half of this is given at noon, and the other at night; or it may be divided into three portions, if the patient prefers. When the hunger becomes excessive, an ounce of meat is added; and, in some extraordinary cases, he begins with six ounces: all other aliment is interdicted. The drink consists of a decoction of two ounces of china-root in two quarts of water boiled into one: this quantity is to be consumed in twenty-four hours; and, if the patient is not satisfied with it, water is added. The extract of chærophylum is taken, night and morning, in the dose of six grains, divided into three pills. After three weeks, one pill only is given, night and morning, and a calomel pill is administered. Towards the seventh week, the patient resumes his habitual labour; is allowed to go out, but is still interdicted the use of strong drinks. This plan is continued three weeks longer; at the end of which time, the original plan of abstinence and the use of the pills is commenced and continued for six weeks. If extensive ulcers of bad character exist, (for small ones require no particular attention,) they are dressed with lint, dipped in a solution of a dram of calomel in a pint of lime-water; if the ulcers become clean, they are dressed with a decoction of china-root, prepared with an ounce of myrrh to each pint; when they manifest a disposition to heal, an ounce of extract of lead is added to it.

This method was first employed on the patients of the hospital at Donveken, near Stockholm, and under the inspection of the College of Medicine. The success was complete; and a commission was appointed, under whose inspection Mr. Osbeck applied his method in the Royal Hospital of St. Seraphin at Stockholm. The result of this treatment has been published in an inaugural dissertation.

The judgment of the commission was,—that the greater part of the patients thus treated were persons who had taken too much mercury, and some others were affected with old scrofulous ulcers: it was considered, however, that the incontestable success of the method, in certain cases, merited a reward. Mr. Osbeck obtained in consequence 3000 crowns and an annual pension of 500. The general result of these experiments is,—that the extract employed, without extreme abstinence, produced little or no effect. The court physician, Schultz, of Schultzenheim, who, notwithstanding his great age, receives with interest, and examines with impartiality, every new discovery, made on himself some experiments with the extract: he took, twice a-day, twelve of Osbeck's pills instead of three, which he recommends. Dr. Lefren went as far as sixty; in neither of these cases was any visible effect perceived. M. de Bierken gave his patient nothing but crumb of bread, with results as satisfactory as those observed by the commission; which seems to prove, that, in the disease treated as venereal in the above instances, much was due to the low diet observed.

---

A paper, containing some singular opinions and original observations, has been lately read to the Medical Society of Paris, by Dr. Serres. It is the result of *an Enquiry into the manner in which Nature remedies some of the organic Injuries of the Brain, particularly those productive of Paralysis.*

The author considers that most cases of paralysis arise from a rupture, or disorganization of the substance of the brain; and that it is to this solution of continuity, that the existence of paralysis, after the effused fluid producing it has become absorbed, is to be attributed. He relates several cases of persons who had recovered from paralytic affections, in whom he had opportunities of ascertaining the state of the brain after death, which took place at different periods subsequently, from various causes. In those instances, re-union of the disunited portions of brain had taken place. The cicatrices were evident; they were of a yellowish tint, and thickly interspersed with capillary blood-vessels. The texture was firmer than that of the rest of the brain; and, on macerating the parts in distilled water for two days, small cells were discovered distributed through them, but which had no communication with each other. In a subject dissected at the Hotel Dieu, a cicatrix was discovered passing longitudinally along the whole extent of one of the hemispheres of the brain of a person who had been paralytic. An interesting case, illustrative of this subject, is related, of a man who had recovered from an attack of apoplexy, followed by hemiplegia of the left side, who, a short time afterwards, fell from a scaffold and broke several of his ribs; the hemiplegia returned, and in a few days he died. A cicatrix was discovered in the centre of the right hemisphere of the brain, having the appearance already described; for some distance from the extremities it was united, but it appeared as if it had been torn asunder in the centre; forming an opening capable of containing a musket-ball. This cavity contained a small quantity of blood.

The author thinks these circumstances will explain the cause why paralysis of the lower limbs is more frequently recovered from, than when the upper extremities are affected. We apprehend he considers, that, as the former most commonly arise from pressure on the spinal cord, on the removal of this pressure, the disease will be removed; while paralysis of the upper extremities, frequently depending on solution of continuity of the brain, can only be recovered from, on re-union of the ruptured parts being effected. M. Serres intends to publish a work on this subject; on the appearance of which, we shall probably enter more fully into the consideration of observations and opinions, which, if true, will lead to some very important improvements in practice.

---

### MEDICAL AND PHILOSOPHICAL INTELLIGENCE.

