# Errata

**Published:** 1818-12

**Authors:** 


— 339, — 35, after 30° add *Reaumur*, or read 100° *Fah.*

